# Cognitive effects of guarana supplementation with maximal intensity cycling

**DOI:** 10.1017/S0007114522002859

**Published:** 2023-07-28

**Authors:** Tom Gurney, Naomi Bradley, Dionisio Izquierdo, Flaminia Ronca

**Affiliations:** 1 University College London, Division of Surgery & Interventional Science, London W1T 7HA, UK; 2 Kingston University School of Life Sciences, Kingston upon Thames KT1 2EE, UK

**Keywords:** Paullinia cupana, Reaction time, Alertness, Cognitive performance, Nutrition

## Abstract

The aim of this study was to investigate the effects of guarana supplementation on cognitive performance before and after a bout of maximal intensity cycling and to compare this to an equivalent caffeine dose. Twenty-five participants completed the randomised double-blind crossover trial by performing cognitive tests with one of three supplements, on three different days: guarana (125 mg/kg), caffeine (5 mg/kg) or placebo (65 mg/kg protein powder). After 30 min of rest, participants performed simple (SRT) and choice reaction time (CRT) tests, an immediate word recall test and Bond–Lader mood scale. This was followed by a cycling *V̇*O_2max_ test, and cognitive tests were then immediately repeated. Guarana supplementation decreased CRT before exercise (407 (sd 45) ms) in comparison with placebo (421 (sd 46) ms, *P* = 0·030) but not caffeine (417 (sd 42) ms). SRT after exercise decreased following guarana supplementation (306 (sd 28) ms) in comparison with placebo (323 (sd 32) ms, *P* = 0·003) but not caffeine (315 (sd 32) ms). Intra-individual variability on CRT significantly improved from before (111·4 (sd 60·5) ms) to after exercise (81·85 (sd 43·1) ms) following guarana supplementation, and no differences were observed for caffeine and placebo (*P* > 0·05). Alertness scores significantly improved following guarana supplementation (63·3 (sd 13·8)) in comparison with placebo (57·4 (sd 13·4), *P* = 0·014) but not caffeine (61·2 (sd 12·8)). There were no changes to *V̇*O_2max_, immediate word recall or any other Bond–Lader mood scales. Guarana supplementation appears to impact several parameters of cognition. These results support the use of guarana supplementation to possibly maintain speed of attention immediately following a maximal intensity exercise test (*V̇*O_2max_).

Guarana (Paullinia cupana) is a native fruit of the Amazonian basin which has been used as a traditional medicine for centuries by indigenous populations^(1)^. The purported biological effects of supplementation are extensive. Reports demonstrate guarana to possess psychoactive and diuretic properties, as well as exhibiting antioxidant and anti-fungal effects^(2)^. Positive stimulating and cognitive effects of guarana have also been reported, with the high caffeine content of its seed (up to 6 % dry weight) being the predominant suggested mechanism of action^(1)^. Indeed, caffeine supplementation possesses a strong efficacy for improvements in a variety of cognitive^(3)^ and physical performances^(4)^. The ubiquitous psychostimulant is predominantly metabolised in the liver by the cytochrome P450 enzyme system^(5)^. Cytochrome P450 isoform 1A2 (CRP1A2) mediates the main caffeine demethylation reaction of N-3 demethylation to paraxanthine, which equates up to 90 % of caffeine demethylation^(5,6)^. The antagonism of adenosine receptors also modulates the rapid movement across the blood–brain barrier which consequently augments dopamine concentrations in the brain^(6)^, resulting in heighted alertness, vigilance, attention and reaction time^(3)^. However, despite the possible ergogenic effects derived from the caffeine content in guarana, reports suggest that the caffeine content is small in typically consumed doses, and in some cases lower than the pharmacological threshold required for humans^(7)^. As such, some studies indicate that additional components may also be the contributing mechanism^(1,7,8)^. Consequently, guarana has become increasingly popular in commercial markets worldwide, particularly in the soft drink and supplementation industry^(1,2)^.

Initial studies conducted by Galduróz and colleagues^(9,10)^ evaluated the acute (1 g/d for 3 d) and chronic (1 g/d for 150 d) cognitive effects of guarana supplementation in young and elderly individuals but found no changes to cognition, sleep or anxiety in either group. No further studies have assessed the long-term effects, but several studies have since reported significant acute cognitive changes following guarana supplementation^(7,11–13)^. Kennedy *et al.* ^(11)^ found both speed of attention and secondary memory were improved by a 75 mg dose of guarana. The increase in speed of attention is reflected in a decrease of reaction time observed in several other studies^(8,11,14,15)^. Haskell *et al.* ^(7)^ corroborated that secondary memory was improved with both 75 and 37·5 mg of guarana. Notably, the lower dose contained less than 9 mg of caffeine, which is considered less than the threshold for pharmacological activity in humans^(7)^. It is therefore speculated that other pharmacokinetic components of guarana (tannins, saponins and flavonoids such as catechins and epicatechins; methylxanthines such as theobromine and theophylline) may be working synergistically with caffeine to enhance the psychoactive effects of the supplement^(1,7–9,11)^. Comparing the effects of guarana to caffeine alone may therefore help elucidate the contribution of the other components within the supplement.

Successful performance in many modern sports necessitates an athlete’s capacity to simultaneously control a variety of physiological and cognitive loads. Such sports include modern pentathlon whereby optimal cognitive functioning is essential during combined events (laser-run)^(16)^ or team sports where many relevant external attentional cues demand quick and accurate decisions. Though it is recognised that moderate exercise improves cognitive performance, higher/maximal intensities are said to worsen cognitive performance^(17)^. Yet to the best of our knowledge, only four studies so far have considered how physical activity may alter the cognitive effects of guarana supplementation, all of which have used moderate intensity exercise (60 % *V̇*O_2max_/peak power and/or rating of perceived exertion (RPE) 13/20) for 30–40 min^(6,8,13,16)^. An interaction between physical activity and guarana supplementation was reported in only one of the studies, where mean reaction time decreased following guarana supplementation in pentathletes after running at an RPE of 13 on the Borg scale^(16)^. Moreover, only one study reported any significant changes to cognition following guarana supplementation alone, and both a decreased reaction time and stable autonomic nervous system (heart rate variability) were reported^(8)^. It is therefore difficult to determine whether the absence of significant changes was the result of combining physical activity and guarana, or due to other extenuating factors.

It is apparent that guarana supplementation affects several areas of cognition which could be highly appealing to athletes who compete in sports that rely heavily on accurate and efficient decision-making processes for successful performance. However, thus far, evidence on how guarana supplementation affects cognitive performance during physical activity is equivocal and limited, particularly as all previous studies have only considered moderate intensities. Increasing the intensity of physical activity is likely to alter the associated fatigue and may therefore interact differently with supplementation compared with moderate intensities of exercise. Therefore, the aim of this study was to evaluate the effects of guarana supplementation on cognitive performance, before and after a bout of maximal intensity exercise. It was hypothesised that guarana supplementation would improve cognitive performance in comparison with caffeine and placebo following a bout of maximal intensity exercise.

## Methods

### Subjects

Twenty-seven physically active participants were recruited from the local university and cycling clubs and two dropped out due to injury or personal extenuating circumstances leaving twenty-five (age: 21 (sd 1) years, height: 172·4 (sd 15·3) cm, mass: 68·5 (sd 12·6) kg). The participants comprised eighteen males and seven females. Participants were considered physically active if they met the UK recommended physical activity guidelines (at least 30 min of moderate intensity activity five times per week). The mean daily caffeine intake of participants was 122 (sd 114) mg. All participants completed both informed consent and physical activity readiness questionnaire forms prior to participation. This study was approved by the UCL Research Ethics Committee in line with the Declaration of Helsinki.

### Supplementation

Employing a double-blind randomised crossover design, participants attended the exercise laboratory on 3-separate days, each 1-week apart. Participants were asked not to eat in the 2-h before testing and not to consume caffeine or alcohol in the 24-h prior to testing. During the intervention, participants were also asked to maintain their regular diet and not to consume any supplements. On each visit, participants drank a different supplement in a randomised order: caffeine (5 mg/kg) (Caffeine, Loughborough, Fisher Scientific), guarana (125 mg/kg) (Guarana Powder, Brighton, The Guarana Company Ltd.) or placebo (65 mg/kg) (protein powder containing undenatured whey protein isolate and instantising agent). The caffeine dose in both caffeine and guarana supplement is in line with the suggested protocol of use by the International Olympic Committee^(4)^. Supplements were diluted in 250 ml of room temperature water and placed into opaque water bottles by an independent member of staff. To minimise olfactory and taste input, participants were instructed to hold their nose while drinking. To enhance the blinding and reduce bias further, participants were initially informed of the broad scope of the study (‘investigating the influences of different supplements on exercise and cognitive function’) but were not informed about the specific supplements and their purported effects. HPLC was performed to determine the caffeine content of the guarana supplement. This was found to be 3·99 %. The guarana dose was then calculated to match the caffeine content of the caffeine supplement given. After drinking the supplement, participants relaxed quietly for 30 min. Following this, participants completed the first set of cognitive tests (see below for protocol).

A *V̇*O_2max_ test was then completed on a cycle ergometer (CareFusion, Vyaire Medical) using a ramp protocol of 25–40 W per minute based on the participants reported level of physical activity. First, a 3-min warm up with no resistance (0 W) was completed. Thereafter, participants started at 0 W and continued until volitional fatigue. The criteria for achieving *V̇*O_2max_ were (1) to attain an respiratory exchange ratio (RER) above 1·1, (2) to achieve a plateau in VO_2_ and (3) heart rate reading (sd 10) beats/min of the age-predicted max^(18)^. The *V̇*O_2max_ was determined by the highest *V̇*O_2_ value that was recorded from the 15-s averages, at this point the peak power output was also recorded. After a 5-min cool down (consisting of unloaded cycling), cognitive tests were repeated.

### Cognitive tests

All cognitive tests were completed on the Computerised Mental Performance Assessment System (COMPASS) (Northumbria University) computer software. This included simple reaction time (SRT), choice reaction time (CRT), secondary memory and mood. For SRT and CRT, 50 stimuli were presented on the screen at random intervals of 1–3 ms. Participants were seated 1 m away from the screen, with their hands placed on the response keys. They were asked to respond as quickly as possible by selecting the appropriate response key (spacebar for SRT, ‘M’ and ‘Z’ keys for CRT), and reaction time was recorded in milliseconds (ms). For SRT, participants had to hit the spacebar as soon as they saw the stimuli, whereas the CRT required a different response depending on the stimulus presented. For example, the screen would show an arrow pointing either left or right. Participants would have to press the ‘Z’ key if the arrow was pointing left, or the ‘M’ key of the arrow was pointing right. The accuracy of responses was recorded as percentage (%). Secondary memory was tested using an immediate word recall test. Fifteen words were presented, each for 1 s, after which participants recalled as many words as possible within 60 s. The number of words and accuracy of recall were recorded. Mood was assessed using the Bond–Lader mood scales^(19)^. At the beginning of each visit, prior to the first set of cognitive tests and any supplementation, researchers explained and familiarised every participant the above cognitive testing battery with a short familiarisation test designed on the COMPASS software, thus dissipating the influence of learning effects^(11)^.

### Intra-individual variability – inconsistency

Inter-individual variability (IIV) refers to short-term fluctuations in behaviour which, when measured across trials of a same session, can be used as an accurate measure of inconsistency (IIV-I), defined as trial-to-trial variability from a single person^(20)^. In line with Costa *et al.* (2019), IIV-I was calculated for SRT and CRT using the sd of item-by-item response times; therefore, each individual’s sd was calculated from the 50 stimuli during the CRT and during the SRT. It has been conceived that inconsistency is determined by higher-order cognitive processes (attentional and executive control) rather than being random noise or result of error variance^(20–22)^ and is therefore worth comparing between supplements to identify their effect on short-term changes in attention.

### Statistical analysis

All variables are expressed as mean values and standard deviation. Statistical analysis was performed using SPSS 26 (IBM). All data sets were analysed for normality using a Shapiro Wilk test. Each outcome measure was analysed using a repeated-measures ANOVA (*α* ≤ 0·05). Where statistically significant differences were identified, a Bonferroni post hoc test was conducted. If parametric assumptions were not met, Friedman’s test was employed. Analysis of the effect of daily caffeine intake on performance was analysed using Pearson correlation coefficients.

## Results

### Simple reaction time

A significant main effect was detected for SRT between supplements (*P* = 0·049, effect size (ES) = 0·11, observed power = 0·58). There was no time effect or supplement × time interaction (*P* > 0·05).

**Fig. 1.**
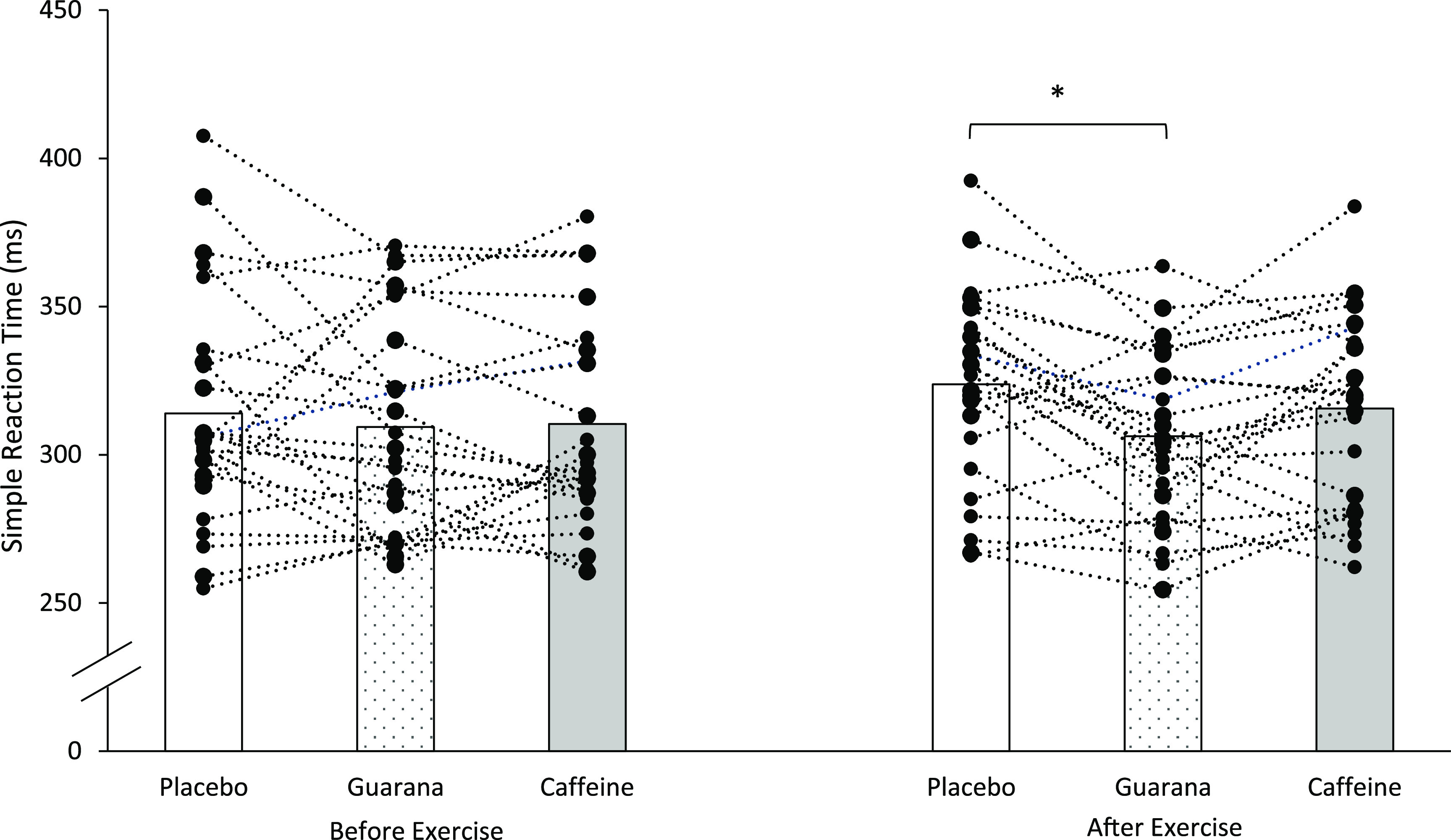
Mean and individual simple reaction time (SRT) before and after exercise. *Signifies a significant difference (*P* ≤ 0·05) between supplements and SRT.

Before exercise, post hoc tests revealed no differences for SRT between supplements (*P* > 0·05), whereas, after exercise, post hoc tests revealed SRT to be significantly lower following guarana supplementation (306 (sd 28) ms) in comparison with placebo (323 (sd 32) ms) (*P* = 0·003). No differences were reported between caffeine (315 (sd 32) ms) *v*. placebo or caffeine *v*. guarana (*P* > 0·05) (Fig. 1).

Therefore, guarana supplementation significantly reduced SRT after exercise compared with placebo, but not compared with caffeine.

### Choice reaction time

A significant main effect was detected for CRT between supplements (*P* = 0·05, ES = 0·11, observed power = 0·57). There was no time effect or supplement × time interaction (*P* > 0·05).

**Fig. 2.**
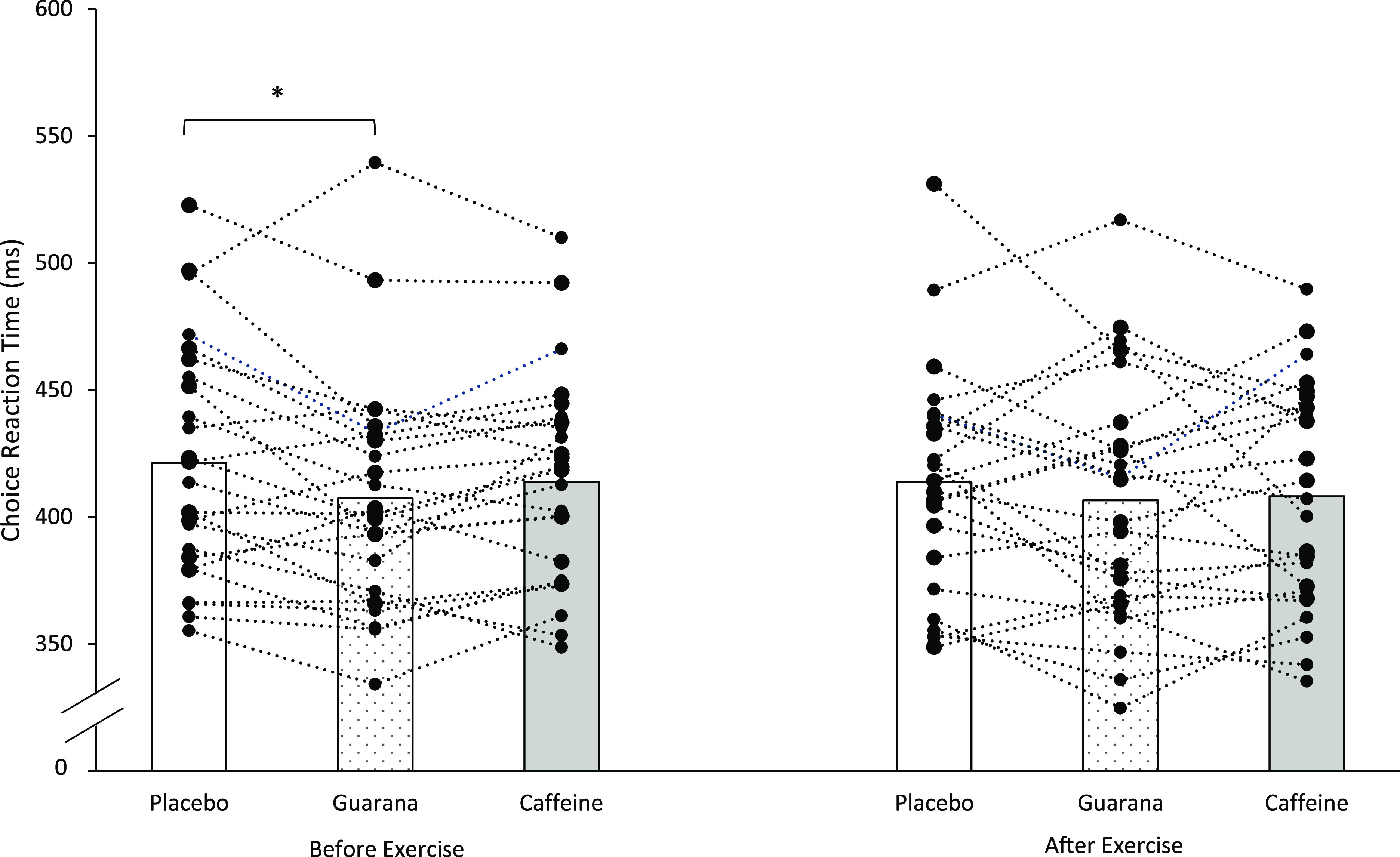
Mean and individual choice reaction time (CRT) before and after exercise. *Signifies a significant difference (*P* ≤ 0·05) between supplements and CRT.

Post hoc tests revealed CRT to be significantly lower before exercise following guarana supplementation (407 (sd 45) ms) in comparison with placebo (421 (sd 46) ms) (*P* = 0·030). No differences were reported between caffeine (417 (sd 42) ms) *v*. placebo or caffeine *v*. guarana before exercise (*P* > 0·05) (Fig. 2). Guarana had the same effect *v*. placebo when only the correct responses were selected (*P* = 0·018).

After exercise (including when only the correct responses were selected), post hoc tests revealed no differences between supplements (*P* > 0·05).

There were no differences in response accuracy percentage between placebo, guarana and caffeine (*P* > 0·05).

Therefore, guarana supplementation significantly reduced CRT response time compared with placebo following 30 min of rest, but there were no differences between supplements after exercise.

### Intra-individual variability – inconsistency

For SRT and CRT IIV-I, there was no significant main effect between supplements or supplement × time interaction (*P* > 0·05).

A significant time effect was detected in SRT (*P* = 0·05, ES = 0·15, observed power = 0·50) where post hoc analysis demonstrated a significant increase in IIV-I from pre- to post-exercise for placebo (*P* = 0·007), whereas for guarana and caffeine there were no differences (*P* > 0·05).

A significant time effect was also detected in CRT (*P* = 0·007, ES = 0·26, observed power = 0·89) where post hoc analysis demonstrated guarana supplementation to significantly decrease IIV-I from pre- to post-exercise (*P* = 0·038). No differences were detected in placebo and caffeine, see Table 1.

**Table 1.** Intraindividual variability inconsistency for both choice (CRT) and simple reaction time (SRT) cognitive tests

**SRT (ms)**	Pre-exercise	Post-exercise	*P* Value
Placebo	54·4 ± 19·9	69·1 ± 21·9	**0·007**
Guarana	50·5 ± 25·4	59·7 ± 29·9	0·127
Caffeine	59·4 ± 25·1	55·5 ± 22·5	0·500
**CRT (ms)**			
Placebo	135·5 ± 91·7	100·1 ± 59·4	0·116
Guarana	111·4 ± 60·5	81·85 ± 43·1	**0·038**
Caffeine	109·3 ± 53·2	86·6 ± 45·65	0·060

**Bold** indicates *P* < 0.05.

Therefore, SRT IIV-I increased significantly after exercise with placebo but not under supplementation with caffeine or guarana, and CRT IIV-I decreased significantly after exercise with guarana only.

### Memory recall

There was no significant main effect, time effect or supplement × time interaction (*P* > 0·05) for memory recall between supplements.

### Bond–Lader mood scales

Parametric assumptions were not met for alert scores; therefore, Friedman’s was employed. Friedman’s reported a significant increase in alert scores following guarana supplementation (63·3 (sd 13·8)) when compared with placebo (57·4 (sd 13·4)) following exercise only (*P* = 0·014). Kendall’s W reported 0·097, indicating a small ES. No differences were reported between caffeine (61·2 (sd 12·8)) *v*. placebo and caffeine *v*. guarana (*P* > 0·05).

There was no significant main effect, time effect or supplement × time interaction (*P* > 0·05) for all other Bond–Lader mood scale variables (content and calm) between supplements.

### Physical performance

No differences in *V̇*O_2max_ were detected between placebo (52·2 (sd 10·7) ml/min per kg), guarana (51·4 (sd 10·0) ml/min per kg) and caffeine (51·7 (sd 9·0) ml/min per g). Equally, no differences in peak power output were detected between placebo (300 (sd 80) Watts), guarana (299 (sd 81) Watts) and caffeine (305 (sd 81) Watts).

### Daily caffeine intake

There was no correlation found between daily caffeine intake and performance on any task or supplement before or after physical activity, with the Pearson correlation coefficient less than 0·08 for all outcome measures.

### Correlations

Spearman’s *ρ* was employed to investigate possible correlations between changes in alert scores and changes in SRT post-exercise (a) and changes in alert scores and changes in CRT IIV-I post-exercise (b). Despite a small trend for (a), the threshold was not met (*P* = 0·058). Similarly, there was no correlation identified for (b) (*P* > 0·05).

## Discussion

The key novel findings of this study demonstrate that guarana supplementation decreased both SRT (after exercise) and CRT (before exercise) in comparison with placebo, supporting previous literature that guarana increases speed of attention^(6–8,11,12,16)^. The supplementation of guarana also improved CRT IIV-I and alert scores, both after exercise. The protective effect against cognitive fatigue was further demonstrated by guarana, whereby SRT IIV-I was maintained following exercise while with placebo it was significantly higher.

The improvement in SRT following guarana supplementation is consistent with previous research and further supports the notion that guarana may possess cognitive stimulant-like effects which therefore enhances information processing speed^(7,8,11,12)^. These findings are also in line with previous investigations that sought to examine the possibility of guarana influencing cognition during/after physical activity whereby significant decreases in mean reaction time and improvements in temporal performance were reported^(6,16)^. IIV-I in the placebo group was significantly higher for SRT following exercise, whereas guarana and caffeine exhibited a plateau with no difference. These results allude to the fact that guarana supplementation can not only improve reaction time but may also do so without hindering cognitive consistency, as measured by trial-to-trial variability which could be caused by physical fatigue. In addition to this, although CRT did not improve after exercise, the IIV-I for CRT following guarana supplementation was significantly reduced (see Table 1), perhaps demonstrating that while participants did not improve in their CRT, they became more consistent with their response times after exercise^(20)^. A mean improvement in consistency after exercise was observed in all conditions but was only significant with guarana supplementation, further suggesting that guarana supplementation in combination with exercise may improve cognitive performance. It is therefore possible that exercise may promote an increase in focus and alertness, which is further augmented by supplementing with a stimulant.

It is important to note that true comparisons to previous literature are difficult as key differences exist in exercise intensity and duration, as well as when cognitive tests commenced. For example, during the Pomportes *et al.* (2017) study, participants were exercising submaximally at 60 % peak power output for 40 min and completed cognitive tasks on the cycle ergometer during this time frame. Guarana supplementation led to an improvement in temporal performance (production) but did not positively influence cognitive control. It is likely that participants would have been in a heightened state of arousal throughout the testing, beyond what is optimal for cognitive performance^(23)^, whereas in this study and previous research, testing was completed after 5 min of recovery or immediately after physical activity^(16)^. Considering participants cooled down for 5 min before being taken to a quiet room for cognitive testing in the current study, this may have allowed for a sufficient amount of recovery before testing. It is also plausible that contrasting results in previous studies are due to differing administration methods. Some studies have combined the consumption of guarana with multivitamin complexes^(6)^ as well as just mouth rinsing^(16)^, while others have employed contrasting caffeine content in comparison with this study^(7,8,12,13)^. In those studies, it is possible that other constituents contained in the multivitamin complexes may have interacted with the effects of guarana.

In the current study, guarana elicited a greater magnitude of effects than the equivalent caffeine dose of 5 mg/kg, suggesting that the combination of caffeine and guarana provides superior simulant-like effects. Despite this, the pharmacokinetics and central nervous system stimulant contributions of caffeine in the current study cannot be ignored. It is well known that caffeine supplementation results in heightened alertness, vigilance, attention and reaction time^(3)^. The rapid absorption of caffeine is mediated by the antagonism of adenosine receptors and the inhibition of phosphodiesterase^(1)^. By competing with and blocking A1 and A2 adenosine receptors, indirectly, the release of norepinephrine, dopamine, acetylcholine and serotonin occurs (Kennedy^(24)^). The release of such neurotransmitters results in excitatory effects which are believed to alter cognitive performance in both habitual and non-habitual consumers^(24)^. This is in keeping with an earlier study by Haskell *et al.* which demonstrated that both habitual consumers and non-consumers had a positive cognitive response from caffeine supplementation at both 75 and 150 mg doses^(25)^. However, daily caffeine intake did not appear to affect performance for any outcome measure in the current study. Guarana’s stimulant-like properties have historically been accredited to the caffeine content of guarana’s seed, but the results from this study add to growing evidence that caffeine cannot be acting exclusively^(1,7)^. Haskell *et al.* ^(7)^ observed guarana to be pharmacologically active at caffeine doses as low as 4·5 mg, below the threshold for activity in humans.

The interaction caffeine has with the other components found within guarana are not yet clear. Evidence suggests that synergistic relationships when co-consumed with other bioactive compounds may result in greater stimulant-like effects. For example, guarana contains traces of several other purine alkaloids (methylxanthines), such as theobromine and theophylline^(24)^. Theobromine, a caffeine derivative, has a higher affinity for A1 receptors in comparison with caffeine, yet it has a longer half-life^(26)^. Studies into these compounds do not suggest a consistent cognitive effect when administered alone but have shown that theobromine appears to act synergistically with caffeine^(27)^. As evidenced by Smit and colleagues^(27)^ whereby caffeine (19 mg) and theobromine (250 mg) combined capsules improved SRT tests, consistent with our current findings. There are also high levels of polyphenols such as tannins (total tannin concentrations estimated between 5 and 14 %) and saponins^(2,24)^. Saponins derived from lychee seeds^(28)^ and ginseng tea leaves^(29)^ have previously demonstrated to possess neuroprotective activities *in vitro* and *in vivo*. The interaction and target of HRAS (HRas proto-oncogene, GTPase), mitogen-activated protein kinase 1) and mitogen-activated protein kinase 8) in the blood–brain barrier via the advanced glycation end products – receptor for advanced glycation end products and PI3K-Akt (phosphatidylinositol 3-kinase – protein kinase B) signalling pathway are the purported mechanisms of action^(29)^. When co-consumed, saponins and tannins may also interact with caffeine and increase the bioavailability of phenols^(24)^. Indeed, there is some promising data to suggest that phenols may improve some cognitive brain functions^(30)^, though the data are not particularly consistent. Although the ingestion of guarana may exert similar stimulant-like effects to caffeine, the presence of tannins and saponins found in guarana may prolong this effect^(31)^. It is therefore plausible to assume that the additional bioactive components of guarana may be acting concomitantly, investigating the psychoactive contribution of each requires more research in order to understand guarana’s full mechanism of action.

The study acknowledges several key limitations. First, the 5-min period between exercise and cognitive tests may not necessarily be translatable and applicable in the field, where athletes or professionals (police/military) are required to make crucial decisions while under physical exertion. Further research should therefore try to eliminate or reduce the time between exertion and testing. Applying relevant cognitive tests to real-world sporting or professional situations would also be pertinent, such tests could place further emphasis on problem solving and higher executive functioning rather than only SRT and CRT. Second, the sample size in the current study was small; therefore, caution must be taken when interpreting the results, particularly when considering the wider applicability of the outcomes. The authors also acknowledge that the study population was specifically young active individuals, some of which came from a cycling club; this therefore impacts the generalisability of the results. It is also plausible to suggest that variations in the erogenicity of caffeine supplementation, and by extension guarana supplementation, may be as a result of inter-individual subtle differences in CYP1A2 and ADORA2A genes^(32)^, measurement of which was beyond the scope of the current study. However, recently there has been increasing conflicting evidence to indicate that caffeine supplementation provides a positive ergogenic response irrespective of the aforementioned genes^(33,34)^. Finally, although supplements were blinded, exit questionnaires were not employed to distinguish whether the participants knew or could guess which supplement they were on. Consequently, this may have influenced the result. Guarana has a distinct taste, but it is unlikely participants would have taken any on its own before and therefore be able to identify it. To combat this, future research could therefore investigate guarana using capsule form.

### Practical and wider applications

This study provides promising evidence that guarana supplementation may be useful by sustaining, or possibly improving, decision making following maximal exertion. This may be particularly pertinent to activities that require bursts of high intensity exercise accompanied by rapid decision making, including team sports, law enforcement and military applications. Previous studies in law enforcement have in fact reported negative effects of stress and exertion on cognitive performance after physically demanding operational tasks^(35)^. Further research could explore the applicability of supplementation strategies in these more specific settings. The magnitude of effect observed with guarana appears to be higher than an equivalent dose of pure caffeine only. Future research should consider the effect of high intensity activity and cognitive performance simultaneously, in conjunction with guarana supplementation, and should consider these effects on higher executive functions that may be applicable to broader fields.

### Conclusion

The findings of this study indicate that acute guarana supplementation leads to significant improvements in both CRT and SRT before and after a maximal bout of exercise, respectively, which is consistent with a heightened feeling of alertness. This improvement, with respect to placebo, is evident in guarana alone, despite a matched dose of caffeine content in the caffeine supplement. These effects appear promising for the use of guarana in sports which require fast response times to be maintained through high intensity physical activity.

## References

[1] Schimpl FC , da Silva JF , de Carvalho Gonçalves JF , et al. (2013) Guarana: revisiting a highly caffeinated plant from the Amazon. J Ethnopharmacol 150, 14–31. 23981847 10.1016/j.jep.2013.08.023

[2] Marques LLM , Ferreira EDF , de Paula MN , et al. (2019) Paullinia cupana: a multipurpose plant-a review. Rev Bras Farmacognosia 29, 77–110.

[3] McLellan TM , Caldwell JA & Lieberman HR (2016) A review of caffeine’s effects on cognitive, physical and occupational performance. Neurosci Biobehav Rev 71, 294–312. 27612937 10.1016/j.neubiorev.2016.09.001

[4] Maughan RJ , Burke LM , Dvorak J , et al. (2018) IOC consensus statement: dietary supplements and the high-performance athlete. Int J Sport Nutr Exerc Metab 28, 104–125. 29589768 10.1123/ijsnem.2018-0020

[5] Grzegorzewski J , Bartsch F , Köller A , et al. (2021) Pharmacokinetics of caffeine: a systematic analysis of reported data for application in metabolic phenotyping and liver function testing. Front Pharmacol 12, 752826. 35280254 10.3389/fphar.2021.752826 PMC8914174

[6] Pomportes L , Brisswalter J , Casini L , et al. (2017) Cognitive performance enhancement induced by caffeine, carbohydrate and guarana mouth rinsing during submaximal exercise. Nutrients 96, 589. 10.3390/nu9060589 PMC5490568 28598402

[7] Haskell CF , Kennedy DO , Wesnes KA , et al. (2007) A double-blind, placebo-controlled, multi-dose evaluation of the acute behavioural effects of guaraná in humans. J Psychopharmacol 21, 65–70. 16533867 10.1177/0269881106063815

[8] Pomportes L , Davranche K , Brisswalter I , et al. (2015) Heart rate variability and cognitive function following a multi-vitamin and mineral supplementation with added guarana (Paullinia cupana). Nutrients 7, 196–208. 10.3390/nu7010196 PMC4303833 25558905

[9] Galduróz JCF & Carlini ED (1994) Acute effects of the Paullinia cupana, ‘Guaraná’ on the cognition of normal volunteers. Sao Paulo Med J 112, 607–611. 7638522 10.1590/s1516-31801994000300007

[10] Galduróz JCF & Carlini ED (1996) The effects of long-term administration of guarana on the cognition of normal, elderly volunteers. Sao Paulo Med J 114, 1073–1078. 8984582 10.1590/s1516-31801996000100003

[11] Kennedy DO , Haskell CF , Wesnes KA , et al. (2004) Improved cognitive performance in human volunteers following administration of guarana (Paullinia cupana) extract: comparison and interaction with Panax ginseng. Pharmacol Biochem Behav 79, 401–411. 15582012 10.1016/j.pbb.2004.07.014

[12] Kennedy DO , Haskell CF , Robertson B , et al. (2008) Improved cognitive performance and mental fatigue following a multi-vitamin and mineral supplement with added guarana (Paullinia cupana). Appetite 50, 506–513. 18077056 10.1016/j.appet.2007.10.007

[13] Veasey RC , Haskell-Ramsay CF , Kennedy DO , et al. (2015) The effects of supplementation with a vitamin and mineral complex with guaraná prior to fasted exercise on affect, exertion, cognitive performance, and substrate metabolism: a randomized controlled trial. Nutrients 7, 6109–6127. 26225993 10.3390/nu7085272 PMC4555111

[14] Scholey A , Bauer I , Neale C , et al. (2013) Acute effects of different multivitamin mineral preparations with and without guaraná on mood, cognitive performance and functional brain activation. Nutrients 5, 3589–3604. 24067387 10.3390/nu5093589 PMC3798923

[15] White DJ , Camfield DA , Maggini S , et al. (2017) The effect of a single dose of multivitamin and mineral combinations with and without guaraná on functional brain activity during a continuous performance task. Nutr Neurosci 20, 8–22. 25259737 10.1179/1476830514Y.0000000157

[16] Pomportes L , Brisswalter J , Hays A , et al. (2019) Effects of carbohydrate, caffeine, and guarana on cognitive performance, perceived exertion, and shooting performance in high-level athletes. Int J Sport Physiol Performance 14, 576–582. 10.1123/ijspp.2017-0865 30300016

[17] Brisswalter J , Collardeau M & René A (2002) Effects of acute physical exercise characteristics on cognitive performance. Sports Med 32, 555–566. 12096929 10.2165/00007256-200232090-00002

[18] Beltz NM , Gibson AL , Janot JM , et al. (2016) Graded exercise testing protocols for the determination of VO2max: historical perspectives, progress, and future considerations. J Sports Med 2016, 3968393. 10.1155/2016/3968393 PMC5221270 28116349

[19] Bond A & Lader M (1974) The use of analogue scales in rating subjective feelings. Br J Med Psychol 47, 211–218.

[20] Costa AS , Dogan I , Schulz JB , et al. (2019) Going beyond the mean: intraindividual variability of cognitive performance in prodromal and early neurodegenerative disorders. Clin Neuropsychol 33, 369–389. 30663511 10.1080/13854046.2018.1533587

[21] Williams BR , Hultsch DF , Strauss EH , et al. (2005) Inconsistency in reaction time across the life span. Neuropsychology 19, 88. 15656766 10.1037/0894-4105.19.1.88

[22] Vasquez BP , Binns MA & Anderson ND (2018) Response time consistency is an indicator of executive control rather than global cognitive ability. J Int Neuropsychol Soc 24, 456–465. 29208077 10.1017/S1355617717001266

[23] Lambourne K & Tomporowski P (2010) The effect of exercise-induced arousal on cognitive task performance: a meta-regression analysis. Brain Res 1341, 12–24. 20381468 10.1016/j.brainres.2010.03.091

[24] Kennedy DO (2019) Phytochemicals for improving aspects of cognitive function and psychological state potentially relevant to sports performance. Sports Med 49, 39–58. 30671903 10.1007/s40279-018-1007-0 PMC6445817

[25] Haskell CF , Kennedy DO , Wesnes KA , et al. (2005) Cognitive and mood improvements of caffeine in habitual consumers and habitual non-consumers of caffeine. Psychopharmacology 179, 813–825 15678363 10.1007/s00213-004-2104-3

[26] Mitchell ES , Slettenaar M , Vd Meer N , et al. (2011) Differential contributions of theobromine and caffeine on mood, psychomotor performance and blood pressure. Physiol Behav 104, 816–822. 21839757 10.1016/j.physbeh.2011.07.027

[27] Smit HJ , Gaffan EA & Rogers PJ (2004) Methylxanthines are the psycho-pharmacologically active constituents of chocolate. Psychopharmacology 176, 412–419. 15549276 10.1007/s00213-004-1898-3

[28] Wang X , Wu J , Yu C , et al. (2017) Lychee seed saponins improve cognitive function and prevent neuronal injury via inhibiting neuronal apoptosis in a rat model of Alzheimer’s disease. Nutrients 9, 105. 28165366 10.3390/nu9020105 PMC5331536

[29] Huang Y , Ding W , Li X , et al. (2022) Memory enhancement effect of saponins from Eleutherococcus senticosus leaves and blood–brain barrier-permeated saponins profiling using a pseudotargeted monitoring strategy. Food Funct 13, 3603–3620. 35262106 10.1039/d1fo03078g

[30] Ammar A , Trabelsi K , Müller P , et al. (2020) The effect of (poly) phenol-rich interventions on cognitive functions and neuroprotective measures in healthy aging adults: a systematic review and meta-analysis. J Clin Med 9, 835. 32204500 10.3390/jcm9030835 PMC7141326

[31] Babu KM , Church RJ & Lewander W (2008) Energy drinks: the new eye-opener for adolescents. Clin Pediatr Emergency Med 9, 35–42.

[32] Southward K , Rutherfurd-Markwick K , Badenhorst C , et al. (2018) The role of genetics in moderating the inter-individual differences in the ergogenicity of caffeine. Nutrients 10, 1352. 30248915 10.3390/nu10101352 PMC6213712

[33] Grgic J , Pickering C , Bishop DJ , et al. (2020) ADORA2A C allele carriers exhibit ergogenic responses to caffeine supplementation. Nutrients 12, 741. 32168870 10.3390/nu12030741 PMC7146260

[34] Glaister M , Chopra K , Pereira de Sena AL , et al. (2021) Caffeine, exercise physiology, and time-trial performance: no effect of ADORA2A or CYP1A2 genotypes. Appl Physiol Nutr Metab 99, 1–11. 10.1139/apnm-2020-0551 33170731

[35] Hope L , Lewinski W , Dixon J , et al. (2012) Witnesses in action: the effect of physical exertion on recall and recognition. Psychol Sci 23, 386–390. 22399414 10.1177/0956797611431463

